# Coarctation of Aorta in Children

**DOI:** 10.7759/cureus.3690

**Published:** 2018-12-05

**Authors:** Arpan R Doshi, Sathish Chikkabyrappa

**Affiliations:** 1 Pediatric Cardiology, Children's Mercy Hospitals and Clinics, Wichita, USA; 2 Pediatric Cardiology, Seattle Children's Hospital, University of Washington School of Medicine, Seattle, USA

**Keywords:** coarctation of aorta, aortic coarctation, pediatric coarctation, cardiac imaging

## Abstract

Coarctation of aorta (CoA) is a discrete narrowing in aorta causing obstruction to the flow of blood. It accounts for 6–8% of all congenital heart diseases. With advances in fetal echocardiography rate of prenatal diagnosis of coarctation of aorta has improved but it still remains a challenging diagnosis to make prenatally. Transthoracic echocardiography is mainstay of making initial diagnosis and routine follow-up. Cardiac magnetic resonance imaging (MRI) and computed tomography (CT) are great advanced imaging tools for two-dimensional and three-dimensional imaging of aortic arch in complex cases. Based on type of coarctation, size of patient, severity of lesion, and associated abnormalities various management options like surgical treatment, transcatheter balloon angioplasty and transcatheter stent implantation are available. There is significant improvement in long-term survival from pre-surgical era to post-surgical era. But, among the postsurgical era patients, the long-term survival has not significantly changed between older and contemporary cohort. Patients with coarctation of aorta need lifelong follow-up event after successful initial intervention.

## Introduction and background

Coarctation of aorta (CoA) can be simply defined as cardiac abnormality resulting in obstruction to the blood flow in the aorta. CoA can occur at any region in the thoracic and abdominal aorta. Most common location for CoA is just distal to the left subclavian artery at the point where ductus arteriosus connects to the aorta. Typically there is presence of medial thickening with “shelf like” tissue protruding in the lumen of aorta from the posterior aortic wall [1, 2]. CoA was first described by Giovanni Morgagni, an Italian anatomist, in the 18th century. First therapeutic surgical intervention was performed for this condition in the year 1944 by Dr. Crafoord [2, 3]. CoA carried a very poor prognosis in the presurgical era with median survival age of mere 31 years [4]. Transcatheter balloon angioplasty was introduced for CoA management in early 1980s by Singer et al. [5]. Later in the decade transcatheter endovascular stent therapy was used for the management of CoA [6]. Now, for about 75 years since first surgical intervention for CoA and significant advances in transcatheter therapy, natural history of this disease has significantly changed. Most of these patients are making it to the adulthood.

## Review

Epidemiology

Congenital heart disease (CHD) accounts for nearly 28% of all major congenital anomalies [7]. The birth incidence of CHD is estimated at eight per 1,000 live births worldwide [8]. CoA accounts for 6–8% of all CHD with an approximate incidence of four per 10,000 live births [9]. It is more common in males than females. CoA is commonly associated with other cardiac and extra-cardiac anomalies. Bicuspid aortic valve, ventricular septal defect, patent ductus arteriosus, transposition of great arteries, etc. are some of the common associated cardiac abnormalities. Bicuspid aortic valve is most frequent associated intracardiac abnormality with prevalence up to 45–62% [4, 10, 11]. CoA is also noted in significant number of patients with Shone complex and other left heart obstructive lesions. CoA is frequently found in patients with genetic syndromes like Turner syndrome and William syndrome. Cramer et al. reported CoA in 18% of their 173 patients with Turner syndrome [12]. William syndrome patients can develop coarctation anywhere in the entire length of aorta, including abdominal aorta.

Pathogenesis

During embryonic period aorta develops from pharyngeal arches and its arterial system. Development of aortic arch starts in third week of gestation and the primordial pharyngeal arch arterial pattern is transformed into the final fetal arterial arrangement during eighth week of gestation. Ventral primitive aorta forms the aortic sac and dorsal primitive aorta forms the descending aorta. Developing pharyngeal arch arterial system connects these two portions. There are six sets of pharyngeal arches that contribute towards development of aortic arch and its branches. It should be noted that all the arches are not present at the same time during fetal period. Primary portion of the aortic arch develops from fourth pharyngeal arch and other arches contribute to development of branch pulmonary arteries, ductus arteriosus and aortic arch branches [9, 13]. Any deviation during this complex developmental process can lead to various aortic anomalies including CoA.

There are three leading developmental theories regarding formation of CoA. First, during development of aortic arch the tissue from ductus arteriosus may get incorporated in the aortic wall where it connects to the descending aorta. As the ductus arteriosus constricts after birth, this tissue in isthmus area constricts causing development of CoA [13, 14]. Second, during fetal life the isthmus area between left subclavian artery and the ductus arteriosus is narrow as this portion carries little blood. This area normally grows in size after birth as the flow through this region increases. Failure of this phenomenon can cause development of CoA [13, 15]. Third, there may be abnormal involution of a small segment of the left dorsal aorta. Later, this narrow region moves cranially with left subclavian artery forming CoA in isthmus region [13].

Clinical presentation

The clinical presentation and exam findings are variable based on patient’s age. Typically earlier presentation corresponds to severe disease.

Younger Children

Newborns and neonates are usually asymptomatic right after birth as patent ductus arteriosus (PDA) helps perfuse lower body irrespective of severity of CoA. Neonates with severe/critical CoA develop signs and symptoms of cardiogenic shock as the ductus arteriosus closes after birth. Clinically, babies may show absent/feeble femoral pulse, delayed capillary refill, feeding problems, decreased responsiveness, metabolic acidosis, mesenteric ischemia, myocardial depression, etc. It is empirical to keep ductus arteriosus open with prostaglandin E1 infusion as soon as the diagnosis of severe/critical CoA is made and further definitive intervention can be performed. Beyond the neonatal period, cardiogenic shock is an unusual presentation but still a possibility during early infancy. Older pediatric patients are usually diagnosed due to weak femoral pulse, upper extremity hypertension, a systolic murmur over upper sternal border with radiation to the back, and upper-lower extremity systolic blood pressure gradient. Newborn pulse oximetry screening is a great tool in detecting cases of critical congenital heart disease in newborns. Although, its utility is limited in patients with pure CoA without presence of PDA (due to lack of mixing/shunting). Newborns with severe/critical coarctation with presence of PDA may have positive results on pulse oximetry screening due to right to left shunting at PDA [16].

Adolescent and Adults

Almost always these patients are diagnosed with CoA during workup of systemic hypertension or heart murmur. Clinical signs may include upper extremity hypertension, weak femoral pulse, arm-leg blood pressure gradient (>20 mmHg is significant), a systolic murmur on the back from flow through the coarctation segment or a continuous murmur from the collateral flow around the coarctation site. Patients may also complain of frequent headaches resulting from systemic hypertension and lower limb claudication from chronic hypoperfusion. If the collateral circulation around the coarctation site is significant then distal pulses may be adequate and arm-leg blood pressure gradient may not be significant.

Hypertension

Systolic hypertension improves and need for antihypertensive medications decreases in almost all patients after successful intervention, but chronic hypertension remains a significant issue in large proportion of patients with CoA. Prevalence of hypertension is lower in patients who are treated during neonatal period and infancy. Overall prevalence of long-term hypertension is noted at 25–68% [17]. Preoperative hypertension is a strong predictor of postoperative long-term hypertension [18]. Proposed mechanisms for late hypertension may include upregulation of renin-angiotensin system, altered vasoreactivity, abnormalities in geometry of aortic arch, dysfunctional baroreceptor mechanism, and abnormal aortic compliance [19, 20]. Ambulatory blood pressure monitor (ABPM) study is a valuable tool in detecting chronic hypertension in these patients.

Diagnostic imaging

Transthoracic echocardiography (TTE) is the preferred diagnostic modality for diagnosis and follow-up of CoA. Fetal echocardiography (FE) has advanced significantly over past couple of decades to allow us to make prenatal diagnosis of CoA and avoid cardiovascular catastrophe after birth. Cardiac MRI (cMRI) and cardiac computed tomography (CT) have emerged as a sophisticated second line of advanced imaging that provides excellent image resolution and anatomical details. Prior to advances in echocardiography, cardiac catheterization was the mainstay for making the diagnosis of CoA. In current era, it is primarily used for interventional purpose.

Fetal Echocardiography

Prenatal diagnosis of CoA helps with parental counselling, delivery planning, avoids postnatal cardiac emergencies, and guides timely management [21]. Prenatal detection of CoA is a challenging diagnosis to make on FE. When there is severe coarctation of aorta and fetus is in the appropriate position for arch imaging it is very straightforward. But majority of times this is not the case and prenatal detection rate of isolated CoA remains low [22]. Some of the following findings on FE should raise the concern for underlying CoA: visualization of discrete narrowing in the region of aortic isthmus, continuous flow across the aortic isthmus on color Doppler, aortic isthmus z-score of less than -2, isthmus-to-ductus arteriosus diameter ratio less than 0.74 on “three vessels and tracheal view”, transverse arch diameter less than 3 mm after 30 weeks of gestation, mitral valve-to-tricuspid valve annulus measurement in four-chamber view less than 0.6, reversal of flow in ductus arteriosus on color Doppler imaging, bidirectional shunting across atrial septum, and disproportional enlargement of right ventricle [23]. When fetus is suspected to have a CoA, serial follow-ups during pregnancy and planned delivery at a tertiary level cardiac center are recommended.

Transthoracic Echocardiography

**Figure 1:**
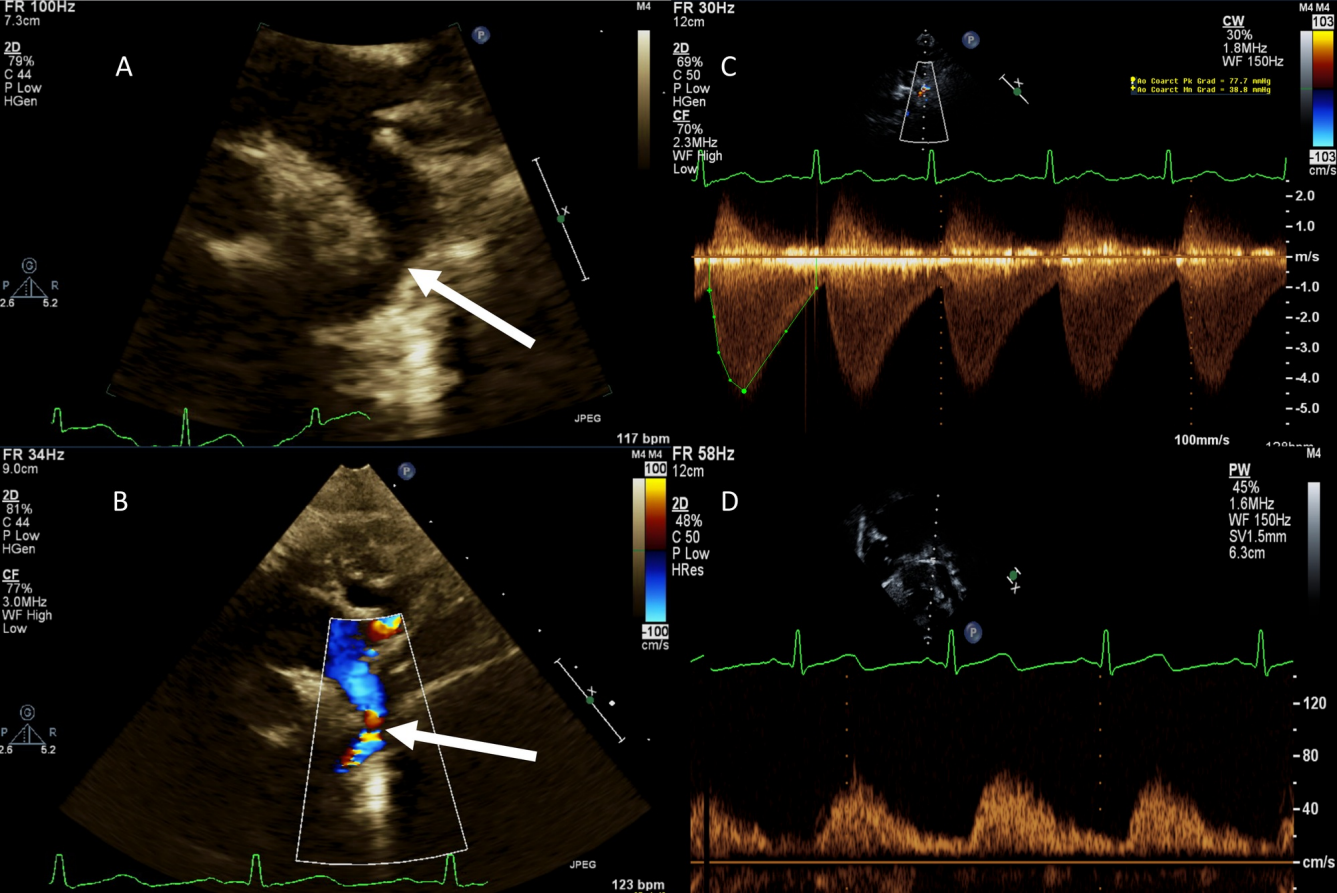
Transthoracic echocardiographic views of coarctation of aorta. (A) Suprasternal sagittal two-dimensional view showing narrowing in the aortic lumen at the isthmus (arrow). (B) Suprasternal sagittal color Doppler imaging showing turbulent flow across the coarctation site (arrow). (C) Continuous wave spectral Doppler imaging across the coarctation segment in suprasternal view. Doppler shows increased flow velocity in systole with continuation of flow in diastole (diastolic run-off). (D) Abnormal Doppler pattern in abdominal aorta in coarctation of aorta. Spectral Doppler shows blunted velocity and systolic upstroke with continuous forward flow.

TTE remains the mainstay of postnatal diagnosis and follow-up for aortic arch anomalies. Goal of TTE is to identify arch anatomy, site of coarctation, determine severity, and assess for associated intracardiac abnormalities [24, 25]. Suprasternal and subcostal views primarily assist with determination of site and severity of the CoA [26]. Two-dimension imaging of arch in suprasternal sagittal view should focus on looking for presence of tissue infolding in the region of aortic isthmus (“posterior shelf”), discrete hypoplasia at the level of aortic isthmus, and diffuse narrowing of any segment of aortic arch (Figure 1). Color Doppler imaging should assess for flow turbulence/increased velocity with diastolic continuation of flow across the suspected coarctation region and presence of ductus arteriosus. Presence of a ductus arteriosus makes it difficult to rule in or out presence of CoA as it connects at the aortic isthmus and changes the flow dynamics at this region of interest [24]. Spectral Doppler shows increased velocity at the coarctation site with “diastolic runoff pattern”. Abdominal aorta Doppler pattern is abnormal with low amplitude spectral Doppler with blunted pulsatility. A complete TTE performed in patients with CoA should also assess for other commonly associated intracardiac abnormalities like bicuspid aortic valve, ventricular septal defect, mitral valve abnormalities, left ventricular hypoplasia, etc. Aortic arch sidedness and branching pattern should also be determined in each patient to assist with surgical planning, if necessary.

Transesophageal Echocardiography

Transesophageal echocardiography is seldom used for primary diagnosis of CoA due to its invasive nature and limited views for arch imaging [27]. It is used for intraoperative imaging in many cases of CoA, typically when other intracardiac abnormalities are addressed.

Computed Tomography

**Figure 2:**
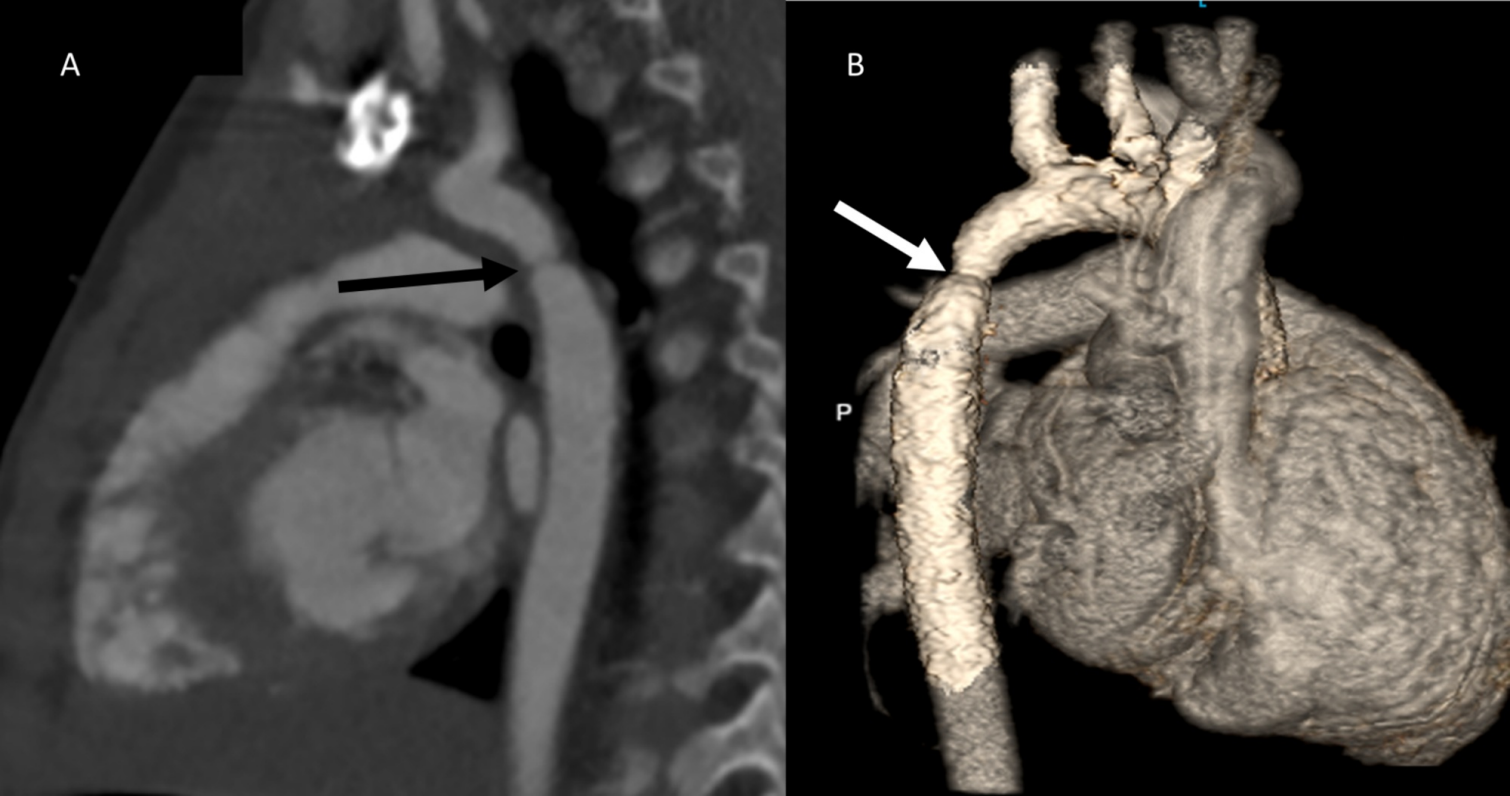
Computed tomography scan imaging of coarctation or aorta. (A) Two-dimensional sagittal reconstruction showing site of discrete narrowing at the level of aortic isthmus (arrow). (B) Three-dimensional reconstruction of computed tomographic angiography showing site of discrete narrowing in the same patient when viewed from posterior aspect (arrow).

CT angiography uses intravenous contrast and ionizing radiation to obtain intracardiac and extracardiac structural data with very high spatial resolution [28]. CT allows to evaluate these structures in two-dimension and also provides ability to reconstruct three-dimensional data (Figure 2). In patients with suboptimal preoperative arch imaging, CT angiography works as a great tool to assist with surgical planning. CT imaging does not produce significant artifact due to metallic objects. This is a great utility for arch imaging in patients with prior coarctation stents [4, 29]. Exposure to ionizing radiation and potential of reaction to contrast material are the primary drawbacks for CT imaging.

Magnetic Resonance Imaging

cMRI is a preferred non-invasive advanced imaging for patients with CoA [27]. cMRI does not include any exposure to ionizing radiation but provides excellent image resolution which makes it ideal for initial imaging and serial follow-ups [30]. cMRI also provides valuable functional and anatomical data regarding other intracardiac structures like aortic valve anatomy, myocardial mass, ventricular function, valve function, etc. cMRI angiography with gadolinium-enhanced contrast provides excellent visualization of extracardiac vasculature and allows for optimal three-dimensional reconstruction as needed. Utilization of phase contrast flow analysis helps to assess peak gradient across the coarctation site [31]. MRI images are susceptible to metallic artifact and hence it is not a good modality of choice in patients with preexisting stents in the region of interest [32].

Electrocardiogram

Electrocardiogram is a typical initial screening test for heart rhythm evaluation and to assess for possible cardiac chamber enlargement. This can be normal or show evidence of left ventricular hypertrophy in patients with CoA. Newborns may show presence of right ventricular hypertrophy on electrocardiogram.

Chest X-Ray

Chest X-ray is helpful at looking for aortic arch sidedness on most patients. Classic “figure of three” sign can be seen on some patients with CoA. This is formed by patent pre-stenotic aortic nob, indentation from stenotic segment, and post-stenotic dilated segment. Patients with long standing unrepaired coarctation develop extensive collateral circulation through dilatation of intercostal arteries. These arteries run on the inferior aspect of the ribs. These patients can show inferior rib notching bilaterally from third to eighth ribs on chest X-ray [33].

Treatment

Surgical repair, transcatheter balloon angioplasty and transcatheter stent implantation are treatment modalities available for management of CoA [34]. Preferred treatment option depends on anatomy of coarctation, age of the patient, size of the patient, and other comorbidities [35]. Some of the widely accepted indications for treatment of native coarctation are as below [2, 4, 27, 36, 37]:

- Non-invasive systolic blood pressure gradient of >20 mmHg between upper and lower limbs

- Peak-to-peak transcatheter gradient of >/= 20 mmHg across the coarctation site

- Peak-to-peak transcatheter gradient of <20 mmHg in the setting of extensive collateral circulation around the coarctation site

- Significant left ventricular hypertrophy

- Left ventricular systolic dysfunction

- Uncontrolled systemic hypertension in the setting of coarctation of aorta

- Abnormal blood pressure response during exercise stress test

Surgery

Since the first surgical repair of CoA in early 1940s by Dr. Crafoord, surgery remains a major treatment option for patients of all age group with CoA [38]. In neonates and young infants surgery is widely accepted as initial intervention of choice for significant CoA [18]. Based on the arch anatomy, site of coarctation and age of the patient various surgical options are available. These techniques include end-to-end anastomosis, extended end-to-end anastomosis, subclavian flap repair, interposition graft, and coarctation resection with prosthetic patch augmentation to mention a few. Vast majority of surgeries for CoA in current era include end-to-end and extended end-to-end anastomosis [39]. Patch augmentation has gone out of favor due to high incidence of aneurysm formation [40]. In theory, subclavian flap repair allows for tension-free repair using autologous tissue and avoids a circumferential suture line decreasing chances of recoarctation. But it does leave residual ductal and coarctation tissue increasing the risk of recoarctation in future. Further subclavian flap repair compromises limb development but does not repair in perceptible functional limitations [41]. Overall, surgical mortality after CoA repair is fairly low [38]. Immediate postoperative course may be complicated by paradoxical hypertension, recurrent laryngeal nerve injury, bleeding, subclavian steal, residual coarctation, etc. Depending on the surgical technique employed and complexity of the lesion some of the long-term complications include recurrent CoA, aneurysm formation, persistent hypertension, and very rarely spinal cord ischemia [42].

Transcatheter Balloon Angioplasty

Transcatheter balloon angioplasty for native CoA was first introduced in the early 1980s [5]. During the procedure a balloon catheter is placed across the coarctation site via retrograde (more common) or antegrade approach. Angiographic sizing of coarctation site as well as aorta proximal and distal to the lesion are performed. Appropriate sized balloon is chosen based on the measurements to dilate the coarctation region [2]. Goal of the procedure is to cause tear in the intima media by overstretching of the narrow vessel site. After this dilatation and creation of tear, aortic wall remodeling is expected to result in long-term resolution of CoA and prevent recoil [43]. Balloon angioplasty is preferred option in older children [34]. It is also preferred choice in younger patients with recoarctation. At present time use of balloon angioplasty in neonates and young infant is mainly reserved in patients with associated ventricular dysfunction to get them stabilized for definitive surgical repair [44]. Its utility as an initial intervention in this very young age group has fallen out of favor due to high recurrence rate and risk of vascular complications [44].

Transcatheter Stent Implantation

Transcatheter stent implantation was introduced in the late 1980s and was widely accepted as a therapeutic measure for patients with CoA in the early 1990s [6]. This is a preferred treatment method for native and recurrent CoA in older children, adolescents and adults [35, 45]. This is technically more challenging when compared to balloon angioplasty and requires larger vascular sheath for access [43]. Once anchored to the aorta, stent provides even distribution of the radial force and provides sustained gradient relief [27, 42, 46]. Results from the Congenital Cardiovascular Interventional Study Consortium (CCISC) and the Coarctation of Aorta Stent Trial (COAST) trials show that stent patients have lower rate of acute complications compared to surgery and balloon angioplasty cohort [34, 35]. But they are more likely to require a planned reintervention for stent dilatation, especially when implanted in younger patient [34]. Acute complications after stent implantation include stent migration, stent embolization, “jailing” of blood vessel, and aortic dissection [34, 35]. Long-term complications include planned reintervention for stent dilatation, neo-intimal proliferation in the stent causing stenosis, stent fracture, and aneurysm [34, 35]. Use of covered stents has decreased the incidence of aneurysms and dissection after stent implantation [47].

Forbes et al. compared safety and efficacy of surgical and transcatheter treatment options for native coarctation at acute interval and at follow-up for patients between the years 2002 and 2009 from 36 institutions [35]. They noted that surgical and stent therapy achieved lower upper-lower extremity blood pressure gradient compared to balloon angioplasty acutely and at short-term follow-up. These differences were disappearing at intermediate follow-up. Stent implantation patients had lower acute complications compared to other two treatment modalities, although stent patients needed more future planned interventions [34]. Younger age at the time of intervention is associated with higher risk of recurrence of CoA [18]. Similar survival rate of about 93% at 10 years, 86% at 20 years, and 74% at 30 years postoperatively is reported in older and contemporary postsurgical cohort [18, 48]. Despite advances in management techniques and medical management the long-term survival has not significantly changed.

## Conclusions

CoA remains most common aortic arch abnormality in children. Key to optimum management of these patients is early detection and timely intervention. Various surgical and transcatheter options are available for management of CoA. Treatment option should be individualized for each patient based on associated factors. Lifelong follow-up is mandatory for these patients to monitor for any late complications.

## References

[1] Campbell M (1970). Natural history of coarctation of the aorta. Br Heart J.

[2] Doshi AR, Syamasundar Rao P (2012). Coarctation of aorta-management options and decision making. Pediat Therapeut.

[3] Kvitting JP, Olin CL (2009). Clarence Crafoord: a giant in cardiothoracic surgery, the first to repair aortic coarctation. Ann Thorac Surg.

[4] Nguyen L, Cook SC (2015). Coarctation of the aorta: strategies for improving outcomes. Cardiol Clin.

[5] Singer MI, Rowen M, Dorsey TJ (1982). Transluminal aortic balloon angioplasty for coarctation of the aorta in the newborn. Am Heart J.

[6] O'Laughlin MP, Perry SB, Lock JE, Mullins CE (1991). Use of endovascular stents in congenital heart disease. Circulation.

[7] van der Linde D, Konings EE, Slager MA, Witsenburg M, Helbing WA, Takkenberg JJ, Roos-Hesselink JW (2011). Birth prevalence of congenital heart disease worldwide: a systematic review and meta-analysis. J Am Coll Cardiol.

[8] Bernier PL, Stefanescu A, Samoukovic G, Tchervenkov CI (2010). The challenge of congenital heart disease worldwide: epidemiologic and demographic facts. Semin Thorac Cardiovasc Surg Pediatr Card Surg Annu.

[9] Singh S, Hakim FA, Sharma A (2015). Hypoplasia, pseudocoarctation and coarctation of the aorta - a systematic review. Heart Lung Circ.

[10] Roos-Hesselink JW, Scholzel BE, Heijdra RJ (2003). Aortic valve and aortic arch pathology after coarctation repair. Heart.

[11] Kappetein AP, Gittenberger-de Groot AC, Zwinderman AH, Rohmer J, Poelmann RE, Huysmans HA (1991). The neural crest as a possible pathogenetic factor in coarctation of the aorta and bicuspid aortic valve. J Thorac Cardiovasc Surg.

[12] Cramer JW, Bartz PJ, Simpson PM, Zangwill SD (2014). The spectrum of congenital heart disease and outcomes after surgical repair among children with Turner syndrome: a single-center review. Pediatr Cardiol.

[13] Moore K, Persaud TVN, Torchia M (2016). The Developing Human: Clinically Oriented Embryology. https://www.elsevier.com/books/the-developing-human/moore/978-0-323-31338-4.

[14] Russell GA, Berry PJ, Watterson K, Dhasmana JP, Wisheart JD (1991). Patterns of ductal tissue in coarctation of the aorta in the first three months of life. J Thorac Cardiovasc Surg.

[15] Rudolph AM, Heymann MA, Spitznas U (1972). Hemodynamic considerations in the development of narrowing of the aorta. Am J Cardiol.

[16] Engel MS, Kochilas LK (2016). Pulse oximetry screening: a review of diagnosing critical congenital heart disease in newborns. Med Devices (Auckl).

[17] Canniffe C, Ou P, Walsh K, Bonnet D, Celermajer D (2013). Hypertension after repair of aortic coarctation--a systematic review. Int J Cardiol.

[18] Brown ML, Burkhart HM, Connolly HM (2013). Coarctation of the aorta: lifelong surveillance is mandatory following surgical repair. J Am Coll Cardiol.

[19] Bhatt AB, Defaria Yeh D (2015). Long-term outcomes in coarctation of the aorta: an evolving story of success and new challenges. Heart.

[20] Ou P, Celermajer DS, Raisky O (2008). Angular (Gothic) aortic arch leads to enhanced systolic wave reflection, central aortic stiffness, and increased left ventricular mass late after aortic coarctation repair: evaluation with magnetic resonance flow mapping. J Thorac Cardiovasc Surg.

[21] Franklin O, Burch M, Manning N, Sleeman K, Gould S, Archer N (2002). Prenatal diagnosis of coarctation of the aorta improves survival and reduces morbidity. Heart.

[22] Familiari A, Morlando M, Khalil A (2017). Risk factors for coarctation of the aorta on prenatal ultrasound: a systematic review and meta-analysis. Circulation.

[23] Rychik J, Tian Z (2011). Fetal Cardiovascular Imaging: A Disease Based Approach. https://www.elsevier.com/books/fetal-cardiovascular-imaging-a-disease-based-approach/rychik/978-1-4160-3172-7.

[24] Goudar SP, Shah SS, Shirali GS (2016). Echocardiography of coarctation of the aorta, aortic arch hypoplasia, and arch interruption: strategies for evaluation of the aortic arch. Cardiol Young.

[25] Lai WW, Mertens LL, Cohen MS, Geva T (2015). Echocardiography in Pediatric and Congenital Heart Disease: From Fetus to Adult. https://www.wiley.com/en-us/Echocardiography+in+Pediatric+and+Congenital+Heart+Disease%3A+From+Fetus+to+Adult-p-9781118337257.

[26] Lai WW, Geva T, Shirali GS (2006). Guidelines and standards for performance of a pediatric echocardiogram: a report from the task force of the Pediatric Council of the American Society of Echocardiography. J Am Soc Echocardiogr.

[27] Dijkema EJ, Leiner T, Grotenhuis HB (2017). Diagnosis, imaging and clinical management of aortic coarctation. Heart.

[28] Nie P, Wang X, Cheng Z, Duan Y, Ji X, Chen J, Zhang H (2012). The value of low-dose prospective ECG-gated dual-source CT angiography in the diagnosis of coarctation of the aorta in infants and children. Clin Radiol.

[29] Gach P, Dabadie A, Sorensen C (2016). Multimodality imaging of aortic coarctation: from the fetus to the adolescent. Diagn Interv Imaging.

[30] Shepherd B, Abbas A, McParland P (2015). MRI in adult patients with aortic coarctation: diagnosis and follow-up. Clin Radiol.

[31] Fogel MA (2010). Principles and Practice of Cardiac Magnetic Resonance in Congenital Heart Disease: Form, Function and Flow. https://www.wiley.com/en-us/Principles+and+Practice+of+Cardiac+Magnetic+Resonance+in+Congenital+Heart+Disease%3A+Form%2C+Function+and+Flow-p-9781444317046.

[32] Bartels LW, Smits HF, Bakker CJ, Viergever MA (2001). MR imaging of vascular stents: effects of susceptibility, flow, and radiofrequency eddy currents. J Vasc Interv Radiol.

[33] Ferguson EC, Krishnamurthy R, Oldham SA (2007). Classic imaging signs of congenital cardiovascular abnormalities. Radiographics.

[34] Adams EE, Davidson WR Jr, Swallow NA, Nickolaus MJ, Myers JL, Clark JB (2013). Long-term results of the subclavian flap repair for coarctation of the aorta in infants. World J Pediatr Congenit Heart Surg.

[35] Forbes TJ, Kim DW, Du W (2011). Comparison of surgical, stent, and balloon angioplasty treatment of native coarctation of the aorta: an observational study by the CCISC (Congenital Cardiovascular Interventional Study Consortium). J Am Coll Cardiol.

[36] Meadows J, Minahan M, McElhinney DB, McEnaney K, Ringel R, COAST Investigators (2015). Intermediate outcomes in the prospective, multicenter coarctation of the aorta stent trial (COAST). Circulation.

[37] Feltes TF, Bacha E, Beekman RH (2011). Indications for cardiac catheterization and intervention in pediatric cardiac disease: a scientific statement from the American Heart Association. Circulation.

[38] Zussman ME, Hirsch R, Herbert C, Stapleton GE (2016). Transcatheter intervention for coarctation of the aorta. Cardiol Young.

[39] Yin K, Zhang Z, Lin Y (2017). Surgical management of aortic coarctation in adolescents and adults. Interact Cardiovasc Thorac Surg.

[40] O'Brien SM, Clarke DR, Jacobs JP (2009). An empirically based tool for analyzing mortality associated with congenital heart surgery. J Thorac Cardiovasc Surg.

[41] Ala-Kulju K, Heikkinen L (1989). Aneurysms after patch graft aortoplasty for coarctation of the aorta: long-term results of surgical management. Ann Thorac Surg.

[42] Cardoso G, Abecasis M, Anjos R, Marques M, Koukoulis G, Aguiar C, Neves JP (2014). Aortic coarctation repair in the adult. J Card Surg.

[43] Gewillig M, Budts W, Boshoff D, Maleux G (2012). Percutaneous interventions of the aorta. Future Cardiol.

[44] Fruh S, Knirsch W, Dodge-Khatami A, Dave H, Pretre R, Kretschmar O (2011). Comparison of surgical and interventional therapy of native and recurrent aortic coarctation regarding different age groups during childhood. Eur J Cardiothorac Surg.

[45] Doshi AR, Rao PS (2013). Development of aortic coarctation following device closure of patent ductus arteriosus. J Invasive Cardiol.

[46] Suarez de Lezo J, Romero M, Pan M (2015). Stent repair for complex coarctation of aorta. JACC Cardiovasc Interv.

[47] Taggart NW, Minahan M, Cabalka AK, Cetta F, Usmani K, Ringel RE, COAST II Investigators (2016). Immediate outcomes of covered stent placement for treatment or prevention of aortic wall injury associated with coarctation of the aorta (COAST II). JACC Cardiovasc Interv.

[48] Cohen M, Fuster V, Steele PM, Driscoll D, McGoon DC (1989). Coarctation of the aorta. Long-term follow-up and prediction of outcome after surgical correction. Circulation.

